# Reflecting on “Marketing communications in health and medicine: perspectives from Willis-Knighton Health System”: understanding the big picture

**DOI:** 10.1186/s12913-020-05607-6

**Published:** 2020-09-15

**Authors:** James K. Elrod, John L. Fortenberry

**Affiliations:** 1 Willis-Knighton Health System, 2600 Greenwood Road, Shreveport, LA 71103 USA; 2 LSU Shreveport, 1 University Place, Shreveport, LA 71115 USA

**Keywords:** Marketing communications, Promotion, Advertising, Hospitals, Healthcare

## Abstract

**Background:**

Willis-Knighton Health System’s special supplement in *BMC Health Services Research*, “Marketing communications in health and medicine: perspectives from Willis-Knighton Health System,” focuses on advertising, public relations, sales promotion, and related communicative avenues, associated theory, and more. Across the supplement’s articles, insights from the institution’s experiences are presented, addressing the components of the marketing communications mix, foundational elements of communication, the patronage process, and the necessity for integrating marketing communications.

**Discussion:**

As an understanding of the big picture is crucial in marketing communications, especially given that many of its components must be effected simultaneously, this particular article takes the insights provided in the supplement and presents them in an operational framework, demonstrating the marketing communications process. This framework concisely summarizes the facets profiled in the associated articles, permitting readers to see how these pieces work in concert with one another in health and medical settings, providing a basic communications structure which healthcare establishments can use to advance their patient engagement initiatives.

**Conclusions:**

Health and medical providers must ensure that they possess a detailed understanding of core marketing communications facets, but as they acquire associated knowledge, they also must direct attention toward understanding the interrelationships between and among these facets, permitting a global perspective of communicative operations. This particular article summarizes insights from Willis-Knighton Health System’s special supplement in *BMC Health Services Research*, providing a pathway toward realizing big picture marketing communications perspectives.

## Background

Willis-Knighton Health System’s special supplement in *BMC Health Services Research*, “Marketing communications in health and medicine: perspectives from Willis-Knighton Health System,” directs attention toward understanding advertising, public relations, sales promotion, and related communicative avenues, associated theory, and more in health services organizations. Communicating effectively with consumers sets the stage for attracting their patronage, capturing vital market share and resulting institutional prosperity [1–7]. The customer acquisition process indeed is ignited by marketing communications, as without such, consumers would remain unaware of the offerings of healthcare providers, hampering their extension of patronage [2, 6, 8–10]. The associated supplement profiles a range of important, related facets of communication, as seen through the lens of Willis-Knighton Health System. Specifically, the collection of articles addresses the components of the marketing communications mix, foundational elements of communication, the patronage process, and the necessity for integrating marketing communications.

Health and medical providers must ensure that they possess a detailed understanding of core marketing communications facets, as presented in the supplement, but as they acquire associated knowledge, they also must direct attention toward understanding the interrelationships between and among these facets, permitting a global perspective of communicative operations. Indeed, a big picture view is crucial in marketing communications, especially given that many of its components must be effected simultaneously, necessitating an understanding of the whole and its parts [2, 5, 10]. To aid healthcare providers in achieving this perspective, this particular article takes the insights provided in the supplement and links them together, providing an operational framework which permits readers to see how the profiled elements work in concert, while also supplying a succinct summary of content, handily concluding the supplement.

## Discussion

Each of the eight core articles included in “Marketing communications in health and medicine: perspectives from Willis-Knighton Health System” supplies information focused specifically on a critical facet of marketing communications, but the supplement, when viewed collectively, offers a sweeping perspective denoting the process of effecting marketing communications in healthcare organizations. Among the insights supplied in the supplement’s five articles which individually profile the marketing communications mix (i.e., advertising, personal selling, sales promotion, public relations, and direct marketing), the supplement references the operative starting point for initiating marketing communications pursuits; namely, the acquisition of sufficient foundational resources to permit successful marketing communications endeavors. Specifically, Willis-Knighton Health System advises establishing the following baseline assets:

- Top leadership support and commitment
- Financial resources sufficient for funding communications activities
- Competent personnel charged with effecting given initiatives
- Formal processes permitting effective planning, implementation, and evaluation of initiatives

These particular resources are required for most any successful organizational endeavor and are needed just the same for marketing communications pursuits. Of course, without requisite foundational assets, failures are virtually guaranteed, necessitating that healthcare providers first direct attention toward securing such before marketing communications efforts begin in earnest. Once secured, health and medical establishments must ensure the adequacy of resources going forward. As such, the periodic evaluation of resources is urged, taking steps, when warranted, to shore up any inadequacies discovered. Complacency is an ever-present threat in organizational life, so vigilance is required in order to avoid associated traps [11, 12]. The ground gained from positive initial marketing communications victories can easily be lost if proper resources are reduced or withdrawn, necessitating efforts to ensure that conveyance pursuits are fueled properly over time [2, 10].

With adequate foundational resources secured, health and medical providers pursuing marketing communications then direct their attention toward putting into place mechanisms which facilitate integration between and among each and every forwarded conveyance, whether it be an advertisement, a direct mail piece, building signage, business cards, or any other communicative device. As conveyed in “Integrated marketing communications: a strategic priority in health and medicine,” the prospects for achieving harmony across marketing conveyances are greatly increased by developing a creative style guide and ensuring associated compliance. By depicting authorized elements of identity, stipulating guidelines for their use, and presenting related strategies, directing the preparation of all marketing communications issued by healthcare providers, this document ensures communicative consistency and cohesion, presenting a unified portrayal of given institutions to their audiences.

With a proficient creative style guide in hand, coupled with the institutional discipline to ensure compliance, attention then can be directed toward building the various elements of identity which will represent given healthcare establishments and their various service offerings. As noted in “Foundational elements of communication in health and medicine: avenues for strengthening the marketing communications mix,” healthcare providers must first direct attention toward the people, places, and things that communicate on behalf of their establishments. Employees, their actions, and their attire; servicescapes, their appearance, and their amenities; logos, their appeal, and their presentation; and related elements speak boldly to the audiences of healthcare organizations [11, 12].

Depending on the quality of their assembly, presentation, and associated maintenance, such artifacts and manifestations have the potential to help or hinder forthcoming outward conveyances of the traditional marketing communications mix, making it imperative for healthcare providers to address them properly prior to formulating and effecting advertising, direct mail, and related components. Also as profiled in “Foundational elements of communication in health and medicine: avenues for strengthening the marketing communications mix,” when healthcare institutions believe their foundational communicators have been addressed properly, Willis-Knighton Health System recommends conducting readiness assessments—evaluative walk-throughs in and around given service environments—to ensure that desired standards and expectations are being realized in practice, increasing the likelihood that audiences will be greeted with satisfaction in their associated interactions. This represents one final opportunity to perfect service experiences prior to the initiation of active, outward marketing communications.

Once foundational elements of communication are in order, healthcare providers turn their attention toward formulating the marketing communications mix. As depicted in the supplement’s associated articles, health and medical establishments examine each of these communicative avenues, selecting one or more believed to be most capable of reaching target audiences. The exact formulation of the marketing communications mix varies between and among healthcare organizations. As institutions, markets, and audiences differ, pat formulas are not possible. Instead, healthcare providers must use their knowledge of the attributes of their particular environments, coupled with insights regarding the components of the marketing communications mix, and devise a formulation anticipated to be best suited for achieving designated communicative goals [2, 10, 13].

Formulating the marketing communications mix indeed requires intensive thought and reflection, with knowledge of strengths and limitations of each component, as profiled in the supplement’s associated articles, being essential for making informed selections. Further, as described in “Response hierarchy models and their application in health and medicine: understanding the hierarchy of effects,” knowledge of the patronage process, especially the particular stage or stages which desired audiences are believed to occupy, is vital when planning communications strategy. The end result of these efforts should yield a game plan for actively pursuing target audiences, notably stipulating the particular avenues of the marketing communications mix that will be used to engage patients.

On launching marketing communications plans, healthcare providers must monitor associated performance, investigating the degree to which communicative goals are being met. As noted in “Advertising in health and medicine: using mass media to communicate with patients,” care especially should be taken to examine the environments in which healthcare institutions are immersed. Environmental circumstances and situations can impact patronage positively or negatively, distorting the perceived performance of marketing communications initiatives. To best assess marketing communications initiatives, triangulation is recommended, monitoring patient inquiries, census levels, and other metrics tied to communicative aims, affording healthcare providers with a range of indicators that can permit informed opinions to be rendered regarding the value delivered. Such an approach is particularly helpful in calculating return on investment, something that is inherently difficult to determine due to the presence of many externalities [14, 15]. Importantly, healthcare providers must carefully record given formulations of the marketing communications mix and results observed, as these insights will aid in formulating future marketing communications initiatives [2, 10, 13]. Having deployed marketing communications and engaged in associated evaluations of inaugural campaigns, healthcare providers are set to plan future marketing communications pursuits, making adjustments as needed to reach designated goals, positively and productively engaging their target audiences.

## Conclusions

The achievement of excellence in marketing communications should be considered mandatory for healthcare providers, as the prospects for acquiring patronage and resulting institutional prosperity largely rest on associated communicative prowess. This requires the acquisition of knowledge about core marketing communications facets, but it also necessitates achieving an understanding of the interrelationships between and among these facets, permitting a global perspective of communicative operations. To aid healthcare providers in gaining this perspective, this particular article summarized insights gleaned from Willis-Knighton Health System’s experiences as profiled across the articles of “Marketing communications in health and medicine: perspectives from Willis-Knighton Health System,” linking together concepts which permit readers to achieve an understanding of the whole and its parts. Ultimately, through this summary presentation, a working operational framework for marketing communications is provided, affording healthcare providers with a basic structure for effecting the communicative process in their given institutions, setting the stage for successful patient engagement initiatives.

## Data Availability

Not applicable.
